# Global, regional, and national burden of heart failure associated with atrial fibrillation

**DOI:** 10.1186/s12872-023-03375-9

**Published:** 2023-07-11

**Authors:** Sanjeewa Kularatna, Amarzaya Jadambaa, Sumudu Hewage, David Brain, Steven McPhail, William Parsonage

**Affiliations:** 1 Australian Centre for Health Services Innovation and Centre for Healthcare Transformation, School of Public Health and Social Work, Queensland University of Technology, Brisbane, Australia; 2 QIMR Berghofer Medical Research Institute, Herston, QLD Australia; 3 Royal Brisbane and Women’s Hospital, Metro North Health, Herston, Australia

**Keywords:** Heart failure, Atrial fibrillation, Global burden, Regional burden, National burden, Population attributable fraction

## Abstract

**Background:**

Heart failure is a leading cause of mortality and morbidity worldwide, and Atrial fibrillation (AF) is among many modifiable risk factors for heart failure. No estimates are available on the magnitude of the burden of heart failure associated with AF, and this study estimated the global, regional, and national burdens associated with AF.

**Methods:**

We used the comparative risk assessment method to estimate the disease burden in terms of prevalence and years lived with disability (YLD). The population-attributable fraction for heart failure and AF was calculated from prevalence estimates of AF and the recalculated relative risks of heart failure associated with AF from a systematic review summarising the longitudinal association between AF and outcomes. The burden of heart failure was retrieved from the Global Burden of Disease database.

**Results:**

Globally, 2.6% (95% uncertainty interval 1.3 to 4.7%) of the burden of heart failure is associated with AF. This was 1.5 (95% UI 0.6 to 3.2) million people in 2019, a 49.8% increase from 1990. The highest prevalence was from South-East Asia, East Asia and Oceania. The highest YLD was estimated for Central Europe, Eastern Europe and Central Asia. High-income countries showed a sharp decline in the age standardised prevalence and YLD rates from 1990 to 2019.

**Conclusion:**

The burden of heart failure associated with AF has increased substantially over the past two decades despite the advances in AF management. However, falling prevalence and YLD rates of heart failure associated with AF in high-income countries over time indicate that reducing this burden is possible.

**Supplementary Information:**

The online version contains supplementary material available at 10.1186/s12872-023-03375-9.

## Background

Heart failure and atrial fibrillation (AF) emerged as two epidemics of cardiovascular disease during the last two decades [1]. In heart failure, a structural and/or functional abnormality of the heart results in elevated intracardiac pressures and/or inadequate cardiac output at rest and/or during exercise [2]. In 2019, approximately 60 million people were living with heart failure globally, a 53% rise from 1990 [3]. Atrial fibrillation is a costly disease. In the United States alone, the annual number of hospitalisations with heart failure as a primary diagnosis increased from 800,000 to more than 1 million from 1990 to 1999 [4]. In 2007 alone, nearly $33 billion was spent on heart failure management in 2007 [5]. This steady increase in the number of patients with heart failure is mostly attributed to better management and survival of patients with myocardial infarction. However, it is important to lessen the increasing burden of heart failure by controlling the risk factors.

Atrial fibrillation (AF), a supraventricular tachyarrhythmia with uncoordinated atrial electrical activation and, consequently, ineffective atrial contraction [6], is a condition associated with AF [7]. Although the causative relationship between AF and heart failure is not yet fully understood, a plausible rationale describing how AF may facilitate the progression of heart failure has been proposed [8]. In AF, the increase in resting heart rate results in shorter diastolic filling time, reducing cardiac output and progressive heart failure. Tachycardia-related myopathy induced by AF can contribute to this further. The first experimental model to examine heart failure and AF demonstrated that rapid atrial pacing led to low output heart failure [9]. Subsequent investigations proved that restoration of sinus rhythm could reverse heart failure [10–13]. More recent reviews also present evidence that AF precedes the later development of heart failure [14, 15]. Collectively, this evidence points out that AF could be considered as a risk factor for heart failure.

Atrial fibrillation negatively influences the prognosis with established heart failure [16] where some AF treatment options are constrained. For example, anti-arrhythmic drugs may be contraindicated or poorly tolerated [6]. Therefore, the co-existence of heart failure and AF present a challenge to clinicians and often results in poor outcomes. This makes prevention of the occurrence or worsening of heart failure a critical goal in the management of AF to improve clinical outcomes and, quality of life and reduce hospital-related costs for these patients. Although the two conditions have been well studied separately, there is a paucity of evidence regarding the epidemiology of heart failure associated with AF. Understanding the magnitude of the burden associated with this dual pathology could provide useful epidemiological evidence on this potentially preventable global public health concern.

The burden of heart failure associated with AF has not been quantified in the Global Burden of Disease (GBD) or other national burdens of disease studies. This knowledge gap prevents prioritised planning and resource allocation for heart failure associated with AF at global, regional, and national levels.

The first aim of our study was to summarise the longitudinal evidence of an association between AF and the later development of heart failure by redistributing study weights according to the quality of studies included in previous systematic reviews and meta-analyses [14]. The second aim was to estimate the burden of heart failure attributable to AF at global, regional, and national levels based on the estimates from the global burden of disease database [17].

## Methods

We used the comparative risk assessment method previously used to estimate ischemic heart disease and stroke burdens attributable to exposure to long working hours [18]. Atrial fibrillation was treated as a risk factor for heart failure using counterfactual estimation and comparative risk assessment methods [19]. This involved comparing the current global health status with the theoretical minimum risk exposure level, assumed to be zero prevalence of AF. Population attributable fractions (PAFs) were estimated for global, regional, and national levels determined by the respective prevalence of AF and the relative risks (RRs) for AF and heart failure.

### Estimation of the population-attributable fraction

#### Prevalence of atrial fibrillation

The prevalence of AF was required as an input parameter in estimating the population-attributable fraction. We retrieved prevalence estimates from the Global Burden of Disease 2019 study [3]. The GBD study estimated the age-standardised prevalence of atrial fibrillation of 0.7 (95% UI 0.6 to 0.9) per 100 000 population using data from systematic reviews and DisMod-MR 2.1, a Bayesian meta-regression tool [20].

#### Relative risk estimates for AF and heart failure

Relative risk (RR) estimates were required as input parameters to calculate the population-attributable fraction. Based on an existing systematic review and meta-analysis [14], we recalculated the RR using a quality effects model [21, 22]. Relative risk estimates from eligible studies published up to June 2016 were included. This existing systematic search identified retrospective (*n* = 1) and prospective (*n* = 5) cohort studies that examined the association between AF and heart failure. Studies reported effect sizes and 95% uncertainty intervals (UIs) comparing those with and without AF. The following details were extracted for each study: study design, country, sample size, gender, method of AF ascertainment, median follow-up period, and measurement of heart failure, as well as effect sizes and 95% UI, which are presented in s1 in the Supplementary material available online.

A quality effects meta-analytic model was used to pool the RR estimates for heart failure. This model is a modified version of the fixed-effects inverse variance method, which gives greater weight to studies of higher quality and lower weight to studies of lower quality. This is achieved by calculating quality scores for each study [21, 22]. Heterogeneity was quantitatively assessed using Cochran’s Q and I^2^ statistics to evaluate whether the pooled studies represent a homogeneous distribution of effect sizes.

The quality of studies was assessed using the Joanna Briggs Institute critical appraisal checklist for cohort studies [23]. This tool has been used in a previous systematic review and meta-analysis [24] and described in more detail in s2 in the Supplementary material. The quality assessment for each study is presented in Table S3 of the supplementary material. Weighted summary measures were computed using MetaXL version 5.3, a plugin package for Microsoft Excel [25]. Relative risks were chosen as the principal summary measure and the meta-analysis was then carried out using adjusted RR estimates. The final pooled RR was presented in tabular format in Table 1 for the RR estimate for AF and heart failure. In further analyses, pooled RR estimates were based on adjusted models, and quality effects models were used to calculate PAFs.

**Table 1:** Characteristics of studies included in the *meta-analyse by Odutayo, Wong *[14]* and recalculated RR using quality-effect model*

**First author, year**	**Study design**	**Method of AF ascertainment**	**Heart failure case definition**	**Number of Participants with AF**	**Number of participants without AF**	**Adjustment**	**Median follow up period (years)**	**Median age (years)**	**Adjusted RR estimates (95%CI)**
Stewart et al. [26]	Prospective follow up cohort	ECG	Heart failure hospitalisation and heart failure death	100	15,306	Age, stroke, chest pain, cholesterol, diastolic blood pressure, cardiothoracic ratio, blood glucose, forced expiratory volume, bronchitis, Q waves, ST segment, left bundle branch block	20	54	3.4 (2.2 to 5.3)
Ruel et al. [27]	Prospective follow up cohort	ECG	Heart failure symptoms, heart failure death, mitral valve repair or replacement	94	754	Age, left ventricular ejection fraction, operative indication, functional mitral regurgitation grade, bioprosthetic implant	5.4	64	4.1 (1.4 to 11.9)
Smit et al. [28]	Prospective follow up cohort	ECG and medical record	NA	121	335	Age, sex, left ventricular ejection fraction, baseline drug therapy, and cumulative right ventricular pacing	2.6	55	2.01 (1.2 to 3.5)
Ruigómez et al. [29]	Prospective follow up cohort	Medical record	any recorded diagnosis of heart failure	831	8226	Age, sex, body mass index, alcohol use, visits to primary care physician, smoking, hypertension, hyperlipidaemia, peripheral vascular disease, venous thromboembolism, chronic obstructive pulmonary disease, diabetes, other cardiac disease	3.6	64	6.4 (5.0 to 8.3)
Conen et al. [30]	Prospective follow up cohort	ECG or medical record	both definite and probable cases of congestive heart failure	1011	33,711	Age, height, body mass index, diabetes, hypertension, systolic blood pressure, hypercholesterolemia, smoking, alcohol consumption, education, randomized treatment assignment, and race/ethnicity	15.4	53	4.2 (3.0 to 5.7)
Andersson et al. [31]	Retrospective follow up cohort	Medical record	Heart failure hospitalisation: ICD 9 and 10: HF 428 (A, B, X) and I50 (0, 1, 9)	9519	12,468	Age, sex, and comorbidities	NA	59	4.6 (4.0 to 5.2)
Pooled RR						demographics, lifestyle risk factors and baseline cardiovascular problems or outcome measures at baseline	4.4 (3.3 to 6.0)^*^ Qi^**^ 17.4 I^2^^***^ 71.3 *P* value for heterogeneity < 0.0001		

*AF* atrial fibrillation, *ECG* electrocardiogram, *HF* heart failure, *ICD RR* relative risk

^*^Forest plot describing the results from individual studies attached in appendix Figure S1

^**^Qi index statistic of the Quality Effects model of the studies

^***^I^2^ statistic for heterogeneity of the studies

#### Calculation of population attributable fraction

The following formula was used to calculate PAFs [32].

$$\mathrm{PAF}=\mathrm{ P}(\mathrm{RR}-1)/\mathrm{P}(\mathrm{RR}-1) +1$$

Where “P” is the prevalence of AF and “RR” is the pooled relative risk of heart failure associated with AF from meta-analyses, adjusted for demographics, lifestyle risk factors and baseline cardiovascular problems or outcome measures at baseline. Population-attributable fractions for heart failure associated with AF at global, regional, and national levels for 1990 and 2019 were calculated using the age-standardised AF prevalence for the respective levels and year. Thus, calculated population-attributable fractions were used to estimate the burden of heart failure associated with AF at corresponding levels and years.

### Computation of the burden of heart failure associated with AF

Population attributable fractions calculated as described above were then applied to estimate the burden of heart failure associated with AF at respective global, regional, and national levels, measured in terms of prevalence and years lived with disability (YLD). All estimates are reported at the global, regional, and national level, by all ages, for both sexes and the year 2019.

We estimated the global burden for heart failure associated with AF for all ages and both sexes for 1990 and 2019, as well as their percentage change for this period. These findings are presented as absolute counts with 95% uncertainty intervals for prevalence and YLDs and further stratified 2019 global burden for heart failure associated with AF by age groups and sex. These results are presented in age-sex pyramids for absolute counts and age-standardised rates for YLDs.

In assessing the regional burden, we calculated the age-standardised prevalence and YLD rates for the burden of heart failure associated with AF, for both sexes, for 2019 and presented in a bar graph for the GBD super regions. In addition, we also analysed the time trends for prevalence and YLDs between 1990 and 2019 for each region, and these are presented in line graphs. The national burden was calculated using the country-level population attributable fraction to the burden of heart failure in terms of absolute counts and age-standardised YLD rated for each individual country and are presented in a world map.

Macro simulation-modelling techniques using MS Excel software were used to calculate uncertainty ranges around point estimates. In this technique, we produced 10,000 iterations of AF prevalence and relative risk for AF and heart failure. This pair-wise distribution yielded a distribution of 10,000 iterations of respective burden values. We used the 2.5^th^ and 97.5^th^ values of this distribution to reflect the lower and upper limits for the 95% uncertainty interval, respectively. This interval reflects the main sources of sampling uncertainty prevalence of AF and relative risks, in the calculations used. All input data and burden are provided in the Supplementary material.

## Results

### The pooled RR estimate based on quality effects model

The result of the meta-analysis for RR estimates for AF and heart failure is presented in Table 1. Individuals with AF had approximately four folds risk [RR = 4.45 (95% UI) 3.28 to 6.04] of developing heart failure compared to individuals without AF.

### Population attributable fraction for heart failure associated with AF

For individuals with AF, the calculated PAF for heart failure for both sexes of all ages were 2.6% (95% UI 1.3 to 4.7%). Overall, the burden of heart failure associated with AF accounted for 0.015% of all-cause YLD (95% UI 0.007 to 0.031%) for both sexes and all ages globally in 2019.

### Global burden of heart failure associated with AF

Despite the reductions in age-standardised rates for prevalence and YLDs, the absolute numbers for heart failure associated with AF have increased from 1990 to 2019 (Table 2). In 2019, 1.5 million (95% UI 0.6 to 3.2) people had developed heart failure associated with AF, and a 51.4% rise compared with 1990. Similarly, the YLD for AF-associated heart failure increased by 53.8% between 1990 and 2019.

**Table 2:** Absolute numbers and age-standardised rates of prevalence and years lived with disability (YLD) due to heart failure associated with atrial fibrillation in 1990, 2019, and percentage change globally for 1990–2019

	**Prevalence**			**YLD**		
	**1990**	**2019**	**Percentage change 1990–2019**	**1990**	**2019**	**Percentage change 1990–2019**
**Atrial Fibrillation**^**a**^						
absolute number, millions	28.3 (21.5 to 36.2)	59.7 (45.7 to 75.3)	52.6%	2.2 (1.4 to 3.2)	4.6 (2.9 to 6.7)	52.2%
ASR per 100,000 population	775.9 (592.4 to 990.8)	743.5 (571.2 to 938.3)	-4.3%	59.63 (37.3 to 87.4)	57.2 (36.1 to 83.0)	-4.3%
**Heart Failure**^**a**^						
absolute number, millions	27.2 (22.2 to 33.3)	56.2 (46.4 to 67.8)	51.6%	2.4 (1.6 to 3.5)	5.0 (3.3 to 7.3)	52.0%
ASR per 100,000 population	765.9 (626.3 to 936.0)	711.9 (591.1 to 858.3)	-7.6%	68.6 (44.3 to 98.7)	63.9 (41.5 to 91.9)	-7.3%
**Heart Failure associated with Atrial Fibrillation**^**b**^						
absolute number, millions	0.7 (0.3 to 1.6)	1.5 (0.6 to 3.2)	49.8%	0.06 (0.02 to 0.17)	0.1 (0.04 to 0.34)	49.8%
ASR per 100,000 population	20.6 (12.8 to 30.7)	18.5 (11.6 to 27.9)	-11.3%	1.8 (1.3 to 2.1)	1.7 (1.2 to 1.9)	-9.9%

*ASR* age-standardised rate, *YLD* years lived with disability

^a^Retrieved from the Global Burden of Diseases database

^b^Calculated within this study

**Fig. 1:**
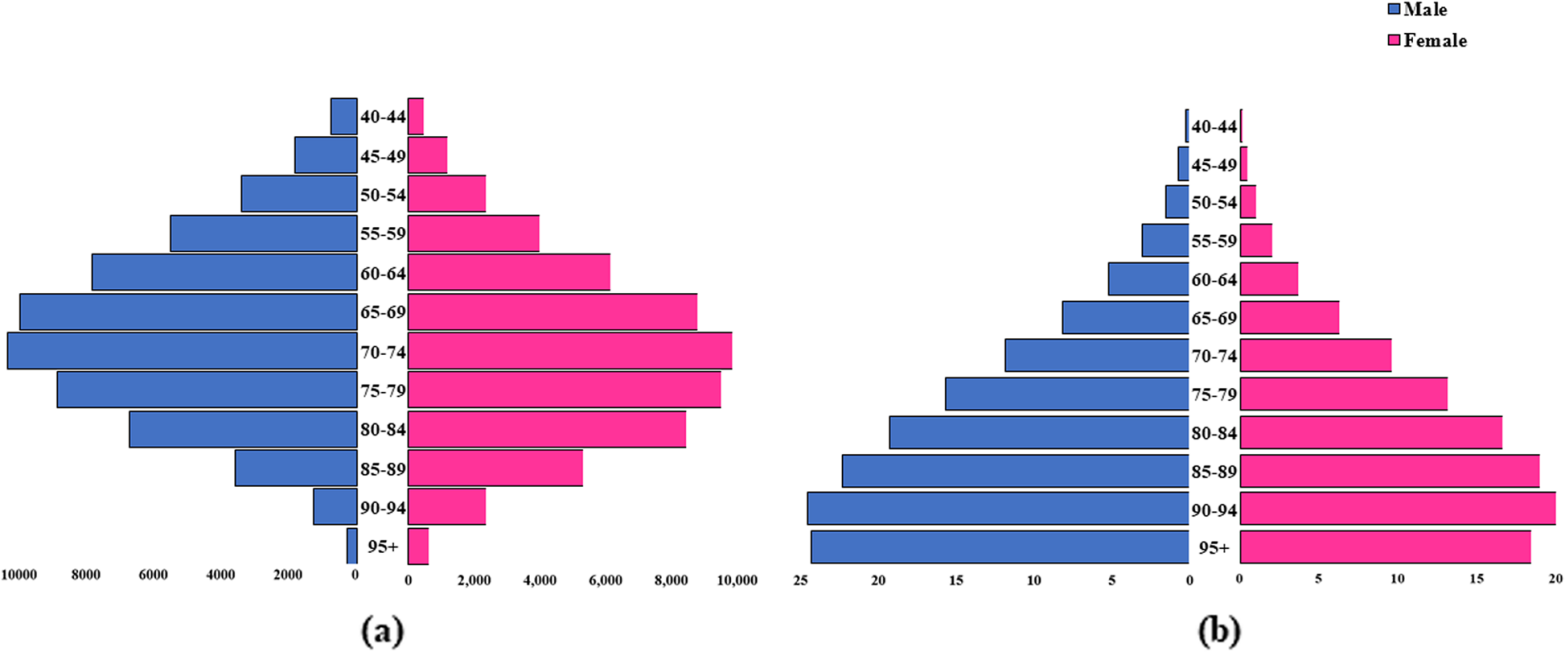
Global YLD (**a**) absolute counts and (**b**) rates for heart failure associated with atrial fibrillation, by age group, by sex, for 2019

The age standardised YLD rates for heart failure associated with AF increased with age and peaked at the age of 90–94 years for both men and women (Fig. 1). The largest burden of YLDs in absolute numbers was in the age groups of 70–74 years for both sexes. This was 10,426 years for males and 9,676 years for females. The supplementary material (Table S4) provides data underpinning these estimates.

#### Regional burden of heart failure associated with AF

**Fig. 2:**
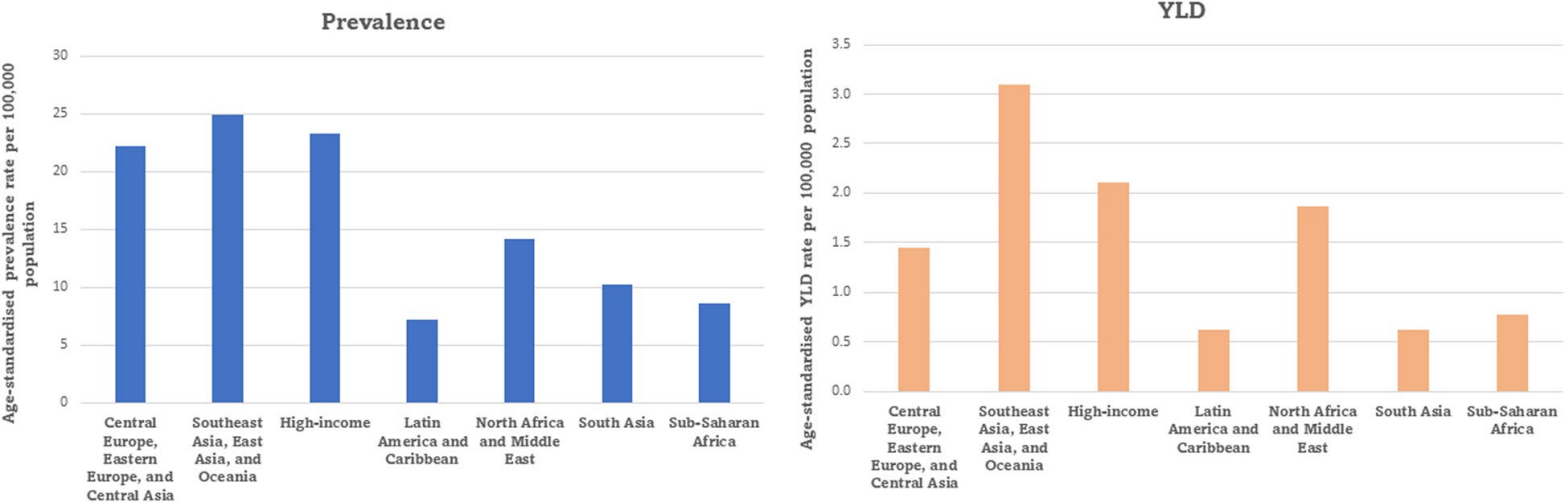
Age standardised prevalence and YLD rates per 100,000 population due to heart failure associated with atrial fibrillation for both sexes, by GBD super region, for 2019

Age-standardised prevalence and YLDs for heart failure associated with AF varied substantially between the GBD super regions (Fig. 2). For 2019, the highest age-standardised prevalence rates per 100,000 population (24.9; 10.3 to 56.3) was estimated for the region of Southeast Asia, East Asia, and Oceania. The lowest prevalence rate of 7.2 (2.9 to 16.6) was estimated for Latin America and the Caribbean. The highest and lowest age standardised YLD rates per 100,000 population were from regions of Central Europe, Eastern Europe and Central Asia (3.1; 1.0 to 8.2) and South Asia (0.62; 0.2 to 1.7). The supplementary material (Table S5) provides data underpinning these estimates.

**Fig. 3:**
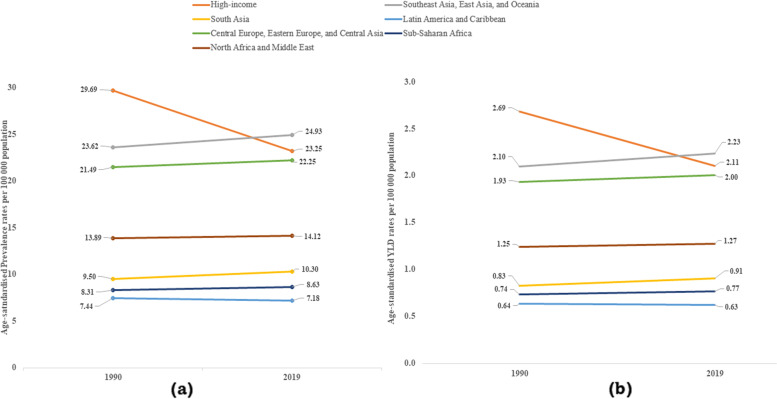
Time trends for age-standardised (**a**) prevalence and (**b**) YLD rates for heart failure associated with atrial fibrillation per 100,000 population in GBD super regions, for both sexes and all ages, 1990–2019. YLD- years lived with disability, GBD—Global Burden of Diseases, Injuries, and Risk Factors Study

We also evaluated the time trends for age-standardised rates for prevalence and YLD for heart failure associated with AF per 100,000 population from 1990 to 2019 for GBD super regions. High-income countries have been able to reduce both the prevalence (from 29.7 to 23.2 per 100,000 population) and YLD (from 2.7 to 2.1 per 100,000 population) during this period, while all other regions show either an increase or a marginal reduction for both prevalence and YLD for heart failure associated with AF (Fig. 3). Data underpinning these estimates are provided in the supplementary material (Tables S6 and S7).

#### National burden of heart failure associated with AF by country

**Fig. 4:**
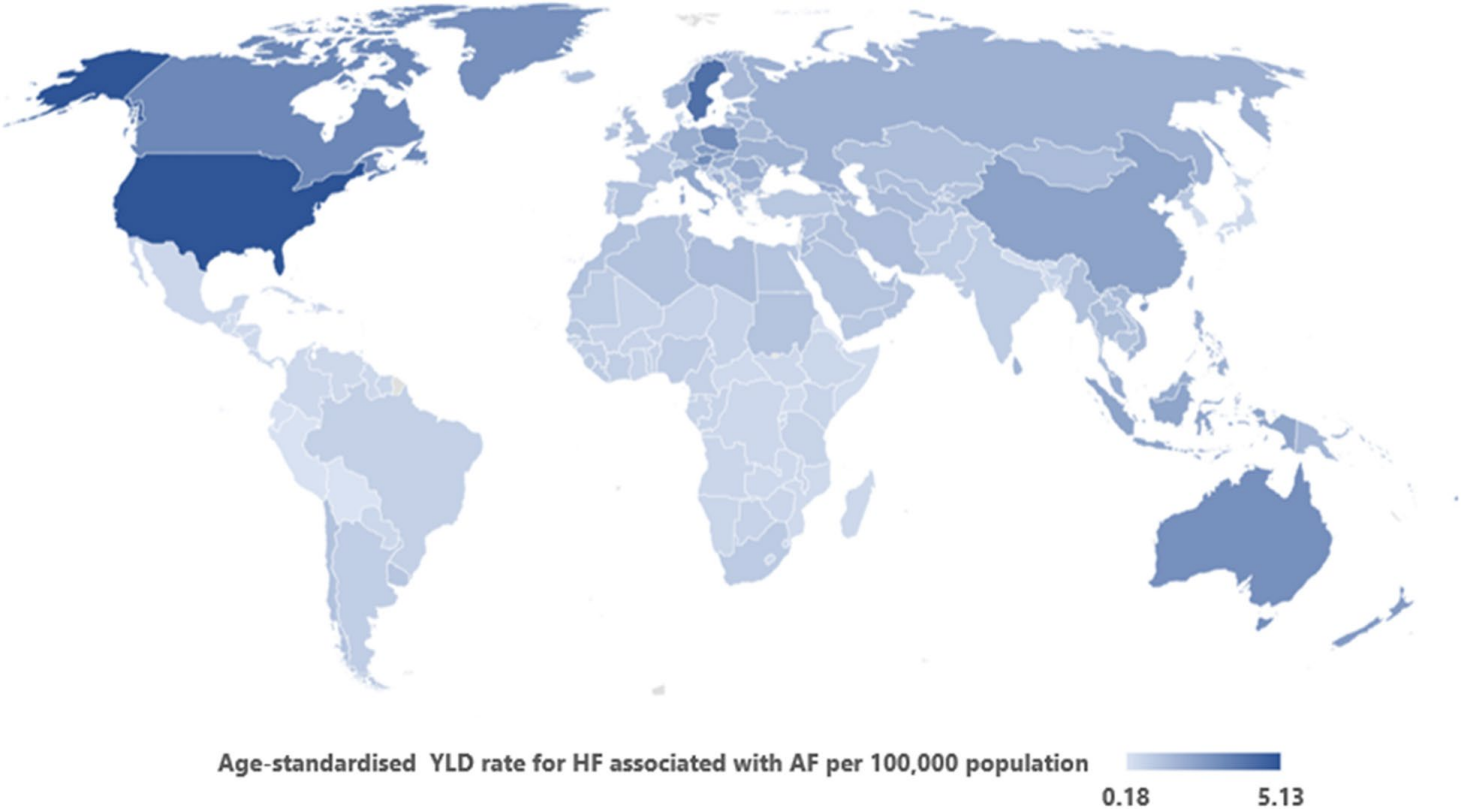
Age-standardised YLD rates for heart failure associated with atrial fibrillation per 100,000 population, for both sexes, by country, for 2019

We calculated the national burden for heart failure associated with AF in terms of age standardised YLD rates per 100,000 population (Fig. 4). In 2019, United States had the highest YLD rates of 5.1 (1.9 to 12.2) per 100,000 population, followed by Sweden (4.1; 1.4 to 10.9) and Canada (3.3; 1.1 to 8.6). Lowest rates were estimated for three Latin American countries-Bolivia (0.18; 0.06 to 0.50), Peru (0.22; 0.07 to 0.61), and Ecuador (0.24; 0.08 to 0.62). All relevant data are provided in the supplementary material (Table S8).

## Discussion

The GBD study estimated that approximately 5 million YLDs for both sexes and all ages were globally attributable to heart failure in 2019 [17]. We estimated the burden of heart failure associated with AF at global, regional, and national levels. Our findings estimate 2.6% (95% UI 1.3 to 4.7) of heart failure is associated with AF and that of all YLD for heart failure, 130,000 (40,000 to 340,000) YLD is associated with AF. This finding highlights the importance of appropriate allocation of healthcare and health research resources to mitigate the disease burden of heart failure and concomitant AF among people with heart failure.

The present study’s findings also indicate that the YLD due to heart failure associated with AF has increased by 49.8% from 1990 to 2019. The increase in these estimates may be attributable to genuine societal health changes, namely, more people living with heart failure and AF. This increase in estimates may also be attributable, in part, to better identification and diagnosis of concomitant AF and heart failure. These findings highlight the potential for reducing the overall disease burden of people living with heart failure through better management of AF. Age-standardised rates were higher among males than females, and rates for both sexes progress with age, consistent with prior studies in the field [33].

Regional estimates for the burden of heart failure associated with AF indicated substantial variation in terms of its prevalence and YLD. Southeast Asia, East Asia and Oceania have the highest prevalence of heart failure associated with AF, even though the burden of AF in this region is much lower than in high-income countries or Central Europe, Eastern Europe and Central Asia [34]. The mechanistic relationship between AF and heart failure is intricate and still not fully comprehended [8]. While some demographic factors such as age and associated comorbidities were accounted for in the analysis, it is likely that there are residual latent traits or unmeasured factors such as the duration, detection, and management of AF that could not be controlled for or explored within the scope of the present study. It is plausible that these unmeasured factors contribute, at least partially, to the inconsistent association between the prevalence of AF and the burden of AF-associated heart failure observed across different regions.

Only high-income countries have been able to reduce the prevalence of heart failure associated with AF between 1990 and 2019. Greater awareness, early detection, improved access to specialist medical care and treatment, and increased investment are likely key factors that have contributed to the reduction in heart failure prevalence, despite the higher prevalence of AF in high-income countries. Gaining a better understanding of the underlying factors behind this reduction in AF-associated heart failure would provide valuable insights and inform the potential for similar improvements to be achieved in other regions.

Regarding the burden of heart failure associated with AF in terms of YLD, high-income countries and Central Europe, Eastern Asia and Central Asia region have the highest estimates. This is consistent with the higher AF prevalence in these regions, but also may reflect the longevity of patients with AF and heart failure in these regions. On the other hand, a reduction in YLD rates from 1990 to 2019 was seen only in high-income countries. This reduction, considered with their similar reduction of prevalence for heart failure associated with AF, is most likely to be due to lowering the occurrence of heart failure in patients with AF, rather than a rise in their mortality rates. However, the burden of fatal cases for heart failure associated with AF could not be estimated as the independent parameter for years of life lost, which reflects fatal outcomes of a disease, are not available for heart failure in the GBD database. The increase in the YLD for other regions could also be due to higher rate of AF diagnosis.

As AF is episodic and often asymptomatic, undiagnosed episodes of AF are common and can remain undiagnosed until or even after the development of complications such as stroke, ischaemic heart disease and heart failure. It is estimated that approximately 10-40% of AF patients remained asymptomatic and undiagnosed even in developed countries such as Japan and the US [35, 36]. Asymptomatic patients with AF carry a higher risk of complications than those with recognised AF who are more likely to receive guideline-directed therapies. Therefore, it is likely that the estimates presented in this paper are conservative, and we are underestimating the true burden of heart failure associated with AF. Screening studies report varying rates of newly diagnosed AF, depending on the screening method and population risk factors [37]. However, research in this field has not yet demonstrated whether people with AF identified from screening practices have the same cardiovascular risks or benefits from standard treatments as people diagnosed with AF through conventional clinical encounters [37].

### Strengths and limitations

There are several strengths of this study. The quality effects model allows quantifying studies according to sample size and by study quality, giving greater weight to studies of high quality. Furthermore, this study controlled for pre-existing cardiovascular disease problems using pooled RRs adjusted for baseline cardiovascular outcomes to quantify PAFs. This protected against the risk of overestimating the disease burden as the continuation of pre-existing cardiovascular disease was adjusted for in the estimates. Finally, PAF estimates provide an opportunity to quantify the burden of heart failure that could be avoided by reducing AF prevalence through effective interventions designed to prevent and treat AF.

The current study also has limitations, which should be considered when interpreting the findings. Firstly, the pathophysiological relationship between AF and heart failure has been only partially elucidated [8], and no firm conclusions have yet been made. Therefore, further exploration of causal mechanisms of action between AF and heart failure remains an important topic for further research. Although this was an important consideration when deciding whether to apply the Comparative Risk Assessment method to estimate the disease burden of heart failure associated with AF, we relied on recent evidence that supports the categorisation of heart failure as a complication of AF [10–15].

Being an episodic disease, a substantial proportion of AF remains undiagnosed [35, 36]. This is associated with difficulty in obtaining reliable epidemiological data and has implications for the use of PAF which may be impacted by underestimates of disease prevalence. Furthermore, epidemiological data reported in prior literature vary depending on the location, study population and method of diagnosis. Therefore, use of PAF most likely underestimates the overall attributable burden of AF, resulting in underestimations of the disease burden from heart failure associated with AF. We tried to minimise the effect of this limitation by performing a meta-analysis of available studies to calculate the relative risk (RR) for AF and heart failure, and by using the pooled RR to calculate the PAF. However, we acknowledge this limitation contributes to noteworthy uncertainty regarding the disease burden estimates.

## Conclusion

Atrial fibrillation poses a significant burden to patients with heart failure and healthcare systems globally. By highlighting the extent of the disease burden of heart failure associated with AF, the present study illustrates the potential for health benefits that could arise from implementing effective programmes to prevent complications of AF. In this study we examined disease burden using YLD; however, healthcare resource use and costs are another important element of societal burden that was beyond the scope of the present study but remains a priority for future research.

## Supplementary Information

**Additional file 1:** **Table S1.** Data extracted from the included studies in SR and MAto estimate the pooled RR for AF and HF. **Table S2.** Quality assessment tool: JBI critical appraisal checklist for cohort studies. **Table S3.** Quality assessment using the JBI critical appraisal checklist for cohort studies. **Table S4.** Absolute numbers and age standardised YLD rates for heart failure associated with atrial fibrillation by age group by sex, for 2019. **Table S5.** Age-standardised prevalence and YLDs for heart failure associated with atrial fibrillation by GBD super regions, for 2019. **Table S6.** Time trends for age-standardised rates for YLD for heart failure associated with AF per 100,000 population from 1990 to 2019 for GBD super regions. **Table S7.** Time trends for age-standardised rates for YLD for heart failure associated with AF per 100,000 population from 1990 to 2019 for GBD super regions. **Table S8.** Age standardised YLD rate for heart failure associated with atrial fibrillation per 100,000 population by country, for 2019. **Figure S1.** Forest plot for the meta-analysis for pooled RR.

## Data Availability

The datasets used and analysed during the systematic review and meta-analyses are available from the corresponding author on request. GBD database accessible publicly at VizHub—GBD Results (healthdata.org) was used for the burden estimates.

## Abbreviations

AF: Atrial fibrillation
ASR: Age-standardised rate
GBD: Global burden of disease study
PAF: Population attributable fraction
RR: Relative risk
UI: Uncertainty interval
YLD: Years lived with disability
