# Supplementary material for: Global, regional, and national burden of heart failure associated with atrial fibrillation
Source: BMC Cardiovasc Disord. 2023 Jul 11;23:345. doi: 10.1186/s12872-023-03375-9 (PMC10334524; doi:10.1186/s12872-023-03375-9)
Supplement: Supplementary file 1 — Additional file 1: Table S1. Data extracted from the included studies in SR and MAto estimate the pooled RR for AF and HF. Table S2. Quality assessment tool: JBI critical appraisal checklist for cohort studies. Table S3. Quality assessment using the JBI critical appraisal checklist for cohort studies. Table S4. Absolute numbers and age standardised YLD rates for heart failure associated with atrial fibrillation by age group by sex, for 2019. Table S5. Age-standardised prevalence and YLDs for heart failure associated with atrial fibrillation by GBD super regions, for 2019. Table S6. Time trends for age-standardised rates for YLD for heart failure associated with AF per 100,000 population from 1990 to 2019 for GBD super regions. Table S7. Time trends for age-standardised rates for YLD for heart failure associated with AF per 100,000 population from 1990 to 2019 for GBD super regions. Table S8. Age standardised YLD rate for heart failure associated with atrial fibrillation per 100,000 population by country, for 2019. Figure S1. Forest plot for the meta-analysis for pooled RR. [file 12872_2023_3375_MOESM1_ESM.pdf]

Global, regional and national burden of heart failure associated with atrial fibrillation.

Supplementary material

Sanjeewa Kularatna[1], Amarzaya Jadambaa[1,2], Sumudu Hewage[1], David Brain[1], Steven McPhail[1,3] William Parsonage[1,4]

[1] Australian Centre for Health Services Innovation and Centre for Healthcare Transformation, School of Public Health and Social Work, Queensland University of Technology, Australia.

[2] QIMR Berghofer Medical Research Institute, Herston, QLD, Australia.

[3] Digital Health and Informatics Directorate, Metro South Health, Queensland, Australia.

[4] Royal Brisbane and Women's Hospital, Metro North Health, Herston, Australia.

Table s1: Data extracted from the included studies in SR and MA (Odutayo et al., 2016) to estimate the pooled RR for AF and HF

First author, year	Location	Study design	Method of atrial fibrillation ascertainment	Heart failure case definition	Number of Participants with AF	Number of participants in control	Adjustment	Median follow up years	Median age (years)	HF cases among AF patients	HF cases among control	Adjusted RR estimates (95%CI)
Stewart et al. (2002)	Scotland	Prospective follow up cohort	ECG	Heart failure hospitalisation and heart failure death	100	15306	Age, stroke, chest pain, cholesterol, diastolic blood pressure, cardiothoracic ratio, blood glucose, forced expiratory volume, bronchitis, Q waves, ST segment, left bundle branch block	20	54	18*	682*	3.4(2.17-5.33)
Ruel et al. (2006)	Canada	Prospective follow up cohort	ECG	Heart failure symptoms, heart failure death, mitral valve repair or replacement	94	754	Age, left ventricular ejection fraction, operative indication, functional mitral regurgitation grade, bioprosthetic implant	5.4	64	NA	NA	4.1(1.41-11.90)
Smit et al. (2006)	Netherlands	Prospective follow up cohort	ECG and medical record	NA	121	335	Age, sex, left ventricular ejection fraction, baseline drug therapy, and cumulative right ventricular pacing	2.6	55	15*	41*	2.01(1.16-3.48)
Ruigómez et al. (2009)	UK	Prospective follow up cohort	Medical record	any recorded diagnosis of heart failure	831	8226	Age, sex, body mass index, alcohol use, visits to primary care physician, smoking, hypertension, hyperlipidaemia, peripheral vascular	3.6	64	125	136	6.4(5.00-8.30)

							disease, venous thromboembolism, chronic obstructive pulmonary disease, diabetes, other cardiac disease					
Conen et al. (2011)	USA	Prospective follow up cohort	ECG or medical record	both definite and probable cases of congestive heart failure	1011	33711	Age, height, body mass index, diabetes, hypertension, systolic blood pressure, hypercholesterolemia, smoking, alcohol consumption, education, randomized treatment assignment, and race/ethnicity	15.4	53	83	252	4.17(3.04-5.71)
Andersson et al. (2014)	Sweden	Retrospective follow up cohort	Medical record	Heart failure hospitalisation: ICD 9 and 10: HF 428 (A, B, X) and I50 (0, 1, 9)	9519	12468	Age, sex, and comorbidities	NA	59	991	297	4.59(4.03-5.22)

*Expected value.

- Andersson, T., Magnuson, A., Bryngelsson, I.-L., Frøbert, O., Henriksson, K. M., Edvardsson, N., & Poçi, D. (2014, 2014/11/15/). Gender-related differences in risk of cardiovascular morbidity and all-cause mortality in patients hospitalized with incident atrial fibrillation without concomitant diseases: A nationwide cohort study of 9519 patients. *International Journal of Cardiology, 177*(1), 91-99. https://doi.org/10.1016/j.ijcard.2014.09.092
- Conen, D., Chae, C. U., Glynn, R. J., Tedrow, U. B., Everett, B. M., Buring, J. E., & Albert, C. M. (2011). Risk of Death and Cardiovascular Events in Initially Healthy Women With New-Onset Atrial Fibrillation. *JAMA, 305*(20), 2080-2087. https://doi.org/10.1001/jama.2011.659

- Odutayo, A., Wong, C. X., Hsiao, A. J., Hopewell, S., Altman, D. G., & Emdin, C. A. (2016). Atrial fibrillation and risks of cardiovascular disease, renal disease, and death: systematic review and meta-analysis. *bmj, 354*.
- Ruel, M., Kapila, V., Price, J., Kulik, A., Burwash, I. G., & Mesana, T. G. (2006). Natural history and predictors of outcome in patients with concomitant functional mitral regurgitation at the time of aortic valve replacement. *Circulation (New York, N.Y.), 114*(1), I541-I546. https://doi.org/10.1161/CIRCULATIONAHA.105.000976
- Ruigómez, A., Johansson, S., Wallander, M.-A., Edvardsson, N., & García Rodríguez, L. A. (2009, 2009/08/14/). Risk of cardiovascular and cerebrovascular events after atrial fibrillation diagnosis. *International Journal of Cardiology, 136*(2), 186-192. https://doi.org/10.1016/j.ijcard.2008.04.050
- Smit, M. D., Van Dessel, P. F., Nieuwland, W., Wiesfeld, A. C., Tan, E. S., Anthonio, R. L., Van Erven, L., Van Veldhuisen, D. J., & Van Gelder, I. C. (2006, Dec). Right ventricular pacing and the risk of heart failure in implantable cardioverter-defibrillator patients. *Heart Rhythm, 3*(12), 1397-1403. https://doi.org/10.1016/j.hrthm.2006.08.006
- Stewart, S., Hart, C. L., Hole, D. J., & McMurray, J. J. V. (2002, 2002/10/01/). A population-based study of the long-term risks associated with atrial fibrillation: 20-year follow-up of the Renfrew/Paisley study. *The American Journal of Medicine, 113*(5), 359-364. https://doi.org/10.1016/S0002-9343(02)01236-6

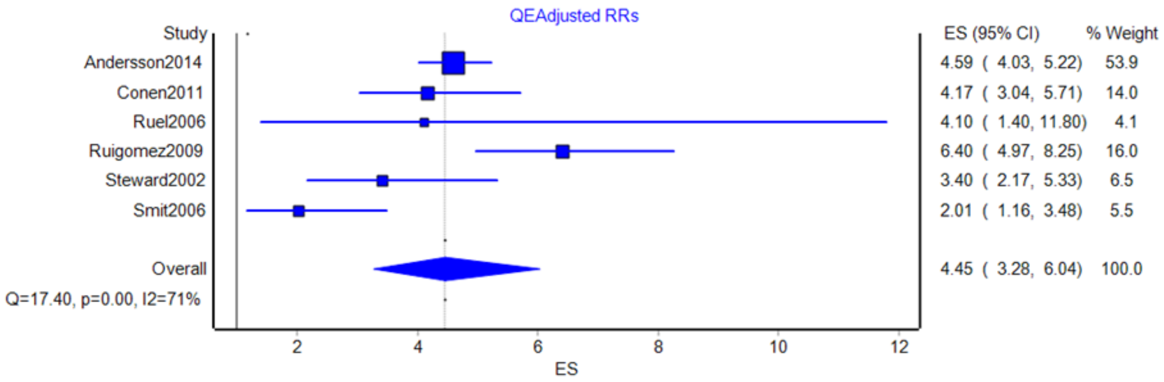

Figure s1. Forest plot for the meta-analysis for pooled RR

Quality assessment:

Quality of studies was assessed using the Joanna Briggs Institute (JBI) critical appraisal checklist for cohort studies (Moola et al., 2020). The total quality score for each study is the sum of the scores for individual assessment items, the maximum quality score for this study was 11. This is converted to a proportional quality score for use in Meta-XL version 5.3 (the total quality score divided by the maximum score possible).

Table s2: Quality assessment tool: JBI critical appraisal checklist for cohort studies

Number of ITEM	Quality criteria	Description of ITEM	Quality score
Item 1	Were the two groups similar and recruited from the same population?	Check the paper carefully for descriptions of participants to determine if patients within and across groups have similar characteristics in relation to exposure (e.g. risk factor under investigation). The two groups selected for comparison should be as similar as possible in all characteristics except for their exposure status, relevant to the study in question. The authors should provide clear inclusion and exclusion criteria that they developed prior to recruitment of the study participants.	• Drawn from the same population = 1<br>• Drawn from a different source/no description= 0
Item 2	Were the exposures measured similarly to assign people to both exposed and unexposed groups?	A high-quality study at the level of cohort design should mention or describe how the exposures were measured. The exposure measures should be clearly defined and described in detail. This will enable reviewers to assess whether or not the participants received the exposure of interest.	•Yes=1<br>•No/Unclear/NA=1

Item 3	Was the exposure measured in a valid and reliable way?	The study should clearly describe the method of measurement of exposure. Assessing validity requires that a 'gold standard' is available to which the measure can be compared. The validity of exposure measurement usually relates to whether a current measure is appropriate or whether a measure of past exposure is needed. Reliability refers to the processes included in an epidemiological study to check repeatability of measurements of the exposures. These usually include intra-observer reliability and inter-observer reliability.	•ECG plus medical record/ECG = 1 • Only medical records=0.5<br>• No description/Unclear/NA= 0
Item 4	Were confounding factors identified?	Confounding has occurred where the estimated intervention exposure effect is biased by the presence of some difference between the comparison groups (apart from the exposure investigated/of interest). Typical confounders include baseline characteristics, prognostic factors, or concomitant exposures (e.g. smoking). A confounder is a difference between the comparison groups, and it influences the direction of the study results. A high-quality study at the level of cohort design will identify the potential confounders and measure them (where possible). This is difficult for studies where behavioral, attitudinal	•Yes=1<br>•No/Unclear/NA=1

		or lifestyle factors may impact on the results.	
Item 5	Were strategies to deal with confounding factors stated?	Strategies to deal with effects of confounding factors may be dealt within the study design or in data analysis. By matching or stratifying sampling of participants, effects of confounding factors can be adjusted for. When dealing with adjustment in data analysis, assess the statistics used in the study. Most will be some form of multivariate regression analysis to account for the confounding factors measured. Look out for a description of statistical methods as regression methods such as logistic regression are usually employed to deal with confounding factors/variables of interest.	•Adjusted for demographics, lifestyle risk factors and baseline cardiovascular problems or outcome measure at baseline = 1<br>•Adjusted for demographics, lifestyle risk factors only = 0.5<br>•Not adjusted=0
Item 6	Were the groups/participants free of the outcome at the start of the study (or at the moment of exposure)?	The participants should be free of the outcomes of interest at the start of the study. Refer to the 'methods' section in the paper for this information, which is usually found in descriptions of participant/sample recruitment, definitions of variables, and/or inclusion/exclusion criteria.	•Yes=1<br>•No/Unclear/NA=1
Item 7	Were the outcomes measured in a valid and reliable way? How was the outcome measured?	Read the methods section of the paper. If for e.g. lung cancer is assessed based on existing definitions or diagnostic criteria, then the answer to this question is likely to be yes. If lung cancer is assessed using observer reported, or self-	• Clinician reported or objective measure (Hospitalisation data, ICD 9 or 10) = 1<br>• Questions from published health surveys/screening instruments or own system

		reported scales, the risk of over- or under-reporting is increased, and objectivity is compromised. Importantly, determine if the measurement tools used were validated instruments as this has a significant impact on outcome assessment validity.<br>Having established the objectivity of the outcome measurement (e.g. lung cancer) instrument, it's important to establish how the measurement was conducted. Were those involved in collecting data trained or educated in the use of the instrument/s? (e.g. radiographers). If there was more than one data collector, were they similar in terms of level of education, clinical or research experience, or level of responsibility in the piece of research being appraised?	/symptoms described/no system/not specified/self-reported = 0
Item 8	Was the follow up time reported and sufficient to be long enough for outcomes to occur?	The appropriate length of time for follow up will vary with the nature and characteristics of the population of interest and/or the intervention, disease or exposure. To estimate an appropriate duration of follow up, read across multiple papers and take note of the range for duration of follow up. The opinions of experts in clinical practice or clinical research may also assist in determining an appropriate duration of follow up. For example, a longer timeframe may be needed to examine	• More than a year=1<br>• Less than a year=0

		the association between occupational exposure to asbestos and the risk of lung cancer. It is important, particularly in cohort studies that follow up is long enough to enable the outcomes. However, it should be remembered that the research question and outcomes being examined would probably dictate the follow up time.	
Item 9	Was follow up complete, and if not, were the reasons to loss to follow up described and explored?	It is important in a cohort study that a greater percentage of people are followed up. As a general guideline, at least 80% of patients should be followed up. Generally, a dropout rate of 5% or less is considered insignificant. A rate of 20% or greater is considered to significantly impact on the validity of the study. However, in observational studies conducted over a lengthy period a higher dropout rate is to be expected. A decision on whether to include or exclude a study because of a high dropout rate is a matter of judgement based on the reasons why people dropped out, and whether dropout rates were comparable in the exposed and unexposed groups.<br>Reporting of efforts to follow up participants that dropped out may be regarded as an indicator of a well conducted study. Look for clear and justifiable description of why people were left out, excluded, dropped out etc. If there is no clear description or	•Completeness good (≥80%), with description of those lost to follow-up = 1<br>•Completeness poor (<80%) or no statement= 0

		a statement in this regard, this will be a 'No'.	
Item 10	Were strategies to address incomplete follow up utilized?	Some people may withdraw due to change in employment, or some may die; however, it is important that their outcomes are assessed.<br>Selection bias may occur because of incomplete follow up. Therefore, participants with unequal follow up periods must be considered in the analysis, which should be adjusted to allow for differences in length of follow up periods. This is usually done by calculating rates which use person-years at risk, i.e., considering time in the denominator.	•Yes=1<br>•No/Unclear/NA=1
Item 11	Was appropriate statistical analysis used?	As with any consideration of statistical analysis, consideration should be given to whether there was a more appropriate alternate statistical method that could have been used. The methods section of cohort studies should be detailed enough for reviewers to identify which analytical techniques were used (in particular, regression or stratification) and how specific confounders were measured.<br>For studies utilizing regression analysis, it is useful to identify if the study identified which variables were included and how they related to the outcome. If stratification was the analytical approach used, were the	• Exposed and non-exposed case numbers and either OR or RR reported = 1<br>• Only OR reported/no description = 0

		strata of analysis defined by the specified variables? Additionally, it is also important to assess the appropriateness of the analytical strategy in terms of the assumptions associated with the approach as differing methods of analysis are based on differing assumptions about the data and how it will respond.	

Table s3. Quality assessment using the JBI critical appraisal checklist for cohort studies.

Studies	Total score out of 11	ITEM 1: Were the two groups similar and recruited from the same population?	ITEM 2: Were the exposures measured similarly to assign people to both exposed and unexposed groups?	ITEM 3: Was the exposure measured in a valid and reliable way?	ITEM 4: Were confounding factors identified?	ITEM 5: Were strategies to deal with confounding factors stated?	ITEM 6: Were the groups/participants free of the outcome at the start of the study (or at the moment of exposure)?	ITEM 7: Were the outcomes measured in a valid and reliable way? How was the outcome measured?	ITEM 8: Was the follow up time reported and sufficient to be long enough for outcomes to occur?	ITEM 9: Was follow up complete, and if not, were the reasons to loss to follow up described and explored?	ITEM 10: Were strategies to address incomplete follow up utilized?	ITEM 11: Was appropriate statistical analysis used?
Andersson et al. (2014)	8.5	1	1	0.5[a]	1	1	1	1	1	0	0	1
Conen et al. (2011)	10.5	1	1	1	1	0.5 [b]	1	1	1	1	1	1
Ruel et al. (2006)	10	1	1	1	1	1	1	0	1	1	1	1
Ruigómez et al. (2009)	8.5	1	1	0.5[a]	1	1	1	1	1	0	0	1

Stewart et al. (2002)	8	1	1	1	1	1	0	1	1	0	0	1
Smit et al. (2006)	8.5	1	1	0.5[a]	1	1	1	1	1	0	0	1

[a] medical record only

[b] adjusted for demographics/lifestyle risk factors only.

References

Andersson, T., Magnuson, A., Bryngelsson, I.-L., Frøbert, O., Henriksson, K. M., Edvardsson, N., & Poçi, D. (2014, 2014/11/15/). Gender-related differences in risk of cardiovascular morbidity and all-cause mortality in patients hospitalized with incident atrial fibrillation without concomitant diseases: A nationwide cohort study of 9519 patients. *International Journal of Cardiology, 177*(1), 91-99. https://doi.org/10.1016/j.ijcard.2014.09.092

Conen, D., Chae, C. U., Glynn, R. J., Tedrow, U. B., Everett, B. M., Buring, J. E., & Albert, C. M. (2011). Risk of Death and Cardiovascular Events in Initially Healthy Women With New-Onset Atrial Fibrillation. *JAMA, 305*(20), 2080-2087. https://doi.org/10.1001/jama.2011.659

Ruel, M., Kapila, V., Price, J., Kulik, A., Burwash, I. G., & Mesana, T. G. (2006). Natural history and predictors of outcome in patients with concomitant functional mitral regurgitation at the time of aortic valve replacement. *Circulation (New York, N.Y.), 114*(1), I541-I546. https://doi.org/10.1161/CIRCULATIONAHA.105.000976

Ruigómez, A., Johansson, S., Wallander, M.-A., Edvardsson, N., & García Rodríguez, L. A. (2009, 2009/08/14/). Risk of cardiovascular and cerebrovascular events after atrial fibrillation diagnosis. *International Journal of Cardiology, 136*(2), 186-192. https://doi.org/10.1016/j.ijcard.2008.04.050

Smit, M. D., Van Dessel, P. F., Nieuwland, W., Wiesfeld, A. C., Tan, E. S., Anthonio, R. L., Van Erven, L., Van Veldhuisen, D. J., & Van Gelder, I. C. (2006, Dec). Right ventricular pacing and the risk of heart failure in implantable cardioverter-defibrillator patients. *Heart Rhythm, 3*(12), 1397-1403. https://doi.org/10.1016/j.hrthm.2006.08.006

Stewart, S., Hart, C. L., Hole, D. J., & McMurray, J. J. V. (2002, 2002/10/01/). A population-based study of the long-term risks associated with atrial fibrillation: 20-year follow-up of the Renfrew/Paisley study. *The American Journal of Medicine, 113*(5), 359-364. https://doi.org/10.1016/S0002-9343(02)01236-6

Table s4. Absolute numbers and age standardised YLD rates for heart failure associated with atrial fibrillation by age group by sex, for 2019.

Age group (years)	Absolute numbers		Age-standardised rate per 100 000 population	
	Male	Female	Male	Female
40-44	776.2	476.1	0.31	0.19
45-49	1845.9	1177.9	0.77	0.50
50-54	3450.2	2325.9	1.59	1.06
55-59	5565.3	3928.8	3.05	2.09
60-64	7892.7	6036.4	5.19	3.76
65-69	10056.5	8654.0	8.14	6.41
70-74	10426.3	9676.7	11.83	9.78
75-79	8948.3	9339.6	15.64	13.37
80-84	6789.0	8299.0	19.27	16.87
85-89	3634.9	5246.1	22.32	19.29
90-94	1302.3	2345.1	24.54	20.30
95+	309.6	654.8	24.26	18.72

YLD – years lived with disability.

Table s5. Age-standardised prevalence and YLDs for heart failure associated with atrial fibrillation by GBD super regions, for 2019.

GBD super region	Age-standardised prevalence (95% UI)	Age-standardised YLD (95% UI)
Southeast Asia, East Asia, and Oceania	24.93 (10.35; 56.35)	3.10 (1.02; 8.20)
High-income countries	23.25 (10.61; 47.72)	2.11 (0.75; 5.25)
Central Europe, Eastern Europe, and Central Asia	22.25 (9.30; 48.76)	1.45 (0.48; 3.76)
North Africa and Middle East	14.12 (5.72; 32.33)	1.87 (0.61; 4.90)
South Asia	10.30 (4.21; 23.43)	0.62 (0.20; 1.66)
Sub-Saharan Africa	8.63 (3.45; 20.25)	0.77 (0.25; 2.09)
Latin America and Caribbean	7.18 (2.91; 16.61)	0.63 (0.20; 1.69)

GBD – global burden of disease study.
YLD – years lived with disability.

Table s6. Time trends for age-standardised rates for YLD for heart failure associated with AF per 100,000 population from 1990 to 2019 for GBD super regions.

GBD super regions	1990	2019	% Change since 1990
High-income	29.69	23.25	-27.72
Southeast Asia, East Asia, and Oceania	23.62	24.93	5.25
South Asia	9.50	10.30	7.69
Latin America and Caribbean	7.44	7.18	-3.62
Central Europe, Eastern Europe, and Central Asia	21.49	22.25	3.41
Sub-Saharan Africa	8.31	8.63	3.68
North Africa and Middle East	13.89	14.12	1.64

GBD – global burden of disease study.
YLD – years lived with disability.

Table s7. Time trends for age-standardised rates for YLD for heart failure associated with AF per 100,000 population from 1990 to 2019 for GBD super regions.

GBD super regions	1990	2019	% Change since 1990
High-income	2.69	2.11	-27.53
Southeast Asia, East Asia, and Oceania	2.10	2.23	5.93
South Asia	0.83	0.91	8.43
Latin America and Caribbean	0.64	0.63	-1.75
Central Europe, Eastern Europe, and Central Asia	1.93	2.00	3.58
Sub-Saharan Africa	0.74	0.77	4.06
North Africa and Middle East	1.25	1.27	2.22

GBD – global burden of disease study.
YLD – years lived with disability.

Table s8. Age standardised YLD rate for heart failure associated with atrial fibrillation per 100,000 population by country, for 2019.

Country/ region	PAF for heart failure and atrial fibrillation (95% UI)			Age standardised rate for YLD for heart failure per 100,000 population (95% UI)			Age standardised YLD rate for heart failure associated with atrial fibrillation, per 100,000 population (95% UI)		
	PAF	LL	UL	YLD	LL	UL	YLD	LL	UL
Afghanistan	1.68%	0.85%	3.11%	60.00	38.36	88.58	1.01	0.33	2.76
Albania	3.32%	1.68%	6.06%	52.74	33.14	76.79	1.75	0.56	4.66
Algeria	1.75%	0.89%	3.27%	72.67	45.65	105.39	1.27	0.41	3.44
American Samoa	2.84%	1.44%	5.23%	83.46	53.73	119.41	2.37	0.77	6.24
Andorra	3.18%	1.62%	5.78%	43.76	27.72	64.00	1.39	0.45	3.70
Angola	1.38%	0.70%	2.57%	44.83	27.54	66.05	0.62	0.19	1.70
Antigua and Barbuda	1.21%	0.62%	2.25%	45.75	28.56	66.73	0.55	0.18	1.50
Argentina	1.85%	0.94%	3.42%	48.55	31.07	70.73	0.90	0.29	2.42
Armenia	3.26%	1.66%	5.93%	50.99	31.70	73.92	1.66	0.53	4.38
Australia	4.40%	2.26%	7.90%	69.59	44.29	102.36	3.06	1.00	8.08
Austria	4.36%	2.28%	7.72%	70.70	47.18	99.30	3.08	1.08	7.66
Azerbaijan	3.20%	1.62%	5.88%	49.99	31.90	73.55	1.60	0.52	4.32
Bahamas	1.23%	0.62%	2.28%	45.14	28.76	66.37	0.55	0.18	1.52
Bahrain	1.84%	0.93%	3.44%	70.67	44.70	103.27	1.30	0.42	3.55
Bangladesh	2.36%	1.19%	4.34%	24.43	15.37	35.24	0.58	0.18	1.53
Barbados	1.26%	0.63%	2.36%	48.26	30.90	70.23	0.61	0.20	1.66
Belarus	3.90%	2.00%	7.13%	42.62	26.86	62.64	1.66	0.54	4.46
Belgium	2.68%	1.40%	4.82%	57.17	37.73	80.77	1.53	0.53	3.89
Belize	1.29%	0.65%	2.43%	51.74	33.02	76.31	0.67	0.22	1.85
Benin	1.45%	0.73%	2.68%	54.40	33.62	81.09	0.79	0.25	2.17
Bermuda	1.30%	0.65%	2.44%	44.93	28.66	65.54	0.58	0.19	1.60
Bhutan	2.50%	1.27%	4.56%	22.84	14.63	33.11	0.57	0.19	1.51

Bolivia	0.54%	0.27%	1.03%	33.59	20.92	48.79	0.18	0.06	0.50
Bosnia and Herzegovina	3.53%	1.80%	6.43%	55.41	35.05	79.63	1.95	0.63	5.12
Botswana	1.50%	0.76%	2.77%	48.37	29.88	72.51	0.73	0.23	2.01
Brazil	1.91%	0.97%	3.53%	42.90	27.66	63.17	0.82	0.27	2.23
Brunei	1.31%	0.67%	2.43%	34.68	22.46	49.16	0.46	0.15	1.19
Bulgaria	3.40%	1.71%	6.22%	52.42	33.78	77.50	1.78	0.58	4.82
Burkina Faso	1.43%	0.72%	2.68%	49.43	30.85	73.70	0.71	0.22	1.97
Burundi	0.84%	0.42%	1.60%	73.65	46.34	111.02	0.62	0.20	1.78
Ivory Coast	1.51%	0.76%	2.81%	58.02	36.26	86.91	0.87	0.28	2.44
Cape Verde	1.55%	0.79%	2.87%	67.71	41.75	100.28	1.05	0.33	2.88
Cambodia	2.57%	1.29%	4.71%	50.68	32.42	74.15	1.30	0.42	3.50
Cameroon	1.54%	0.78%	2.86%	49.74	31.30	74.28	0.77	0.24	2.13
Canada	4.54%	2.38%	8.09%	72.81	46.59	106.48	3.31	1.11	8.61
Central African Republic	1.33%	0.67%	2.46%	37.07	22.46	55.62	0.49	0.15	1.37
Chad	1.43%	0.73%	2.67%	50.97	31.74	75.83	0.73	0.23	2.03
Chile	2.10%	1.06%	3.88%	60.61	38.90	89.79	1.27	0.41	3.48
China	2.61%	1.33%	4.78%	92.59	59.89	135.06	2.42	0.79	6.45
Colombia	1.29%	0.65%	2.40%	40.11	25.25	59.03	0.52	0.16	1.41
Comoros	0.78%	0.39%	1.46%	77.47	48.38	117.03	0.60	0.19	1.70
Congo	1.42%	0.71%	2.65%	48.21	29.72	71.63	0.68	0.21	1.90
Cook Islands	2.75%	1.40%	5.07%	85.99	54.89	126.62	2.37	0.77	6.42
Costa Rica	1.33%	0.67%	2.49%	39.86	25.36	58.46	0.53	0.17	1.45
Croatia	3.00%	1.59%	5.35%	38.94	26.33	55.19	1.17	0.42	2.95
Cuba	1.22%	0.62%	2.26%	45.23	28.71	66.71	0.55	0.18	1.51
Cyprus	2.99%	1.52%	5.49%	42.03	27.40	60.57	1.25	0.42	3.32
Czech Republic	3.92%	1.99%	7.14%	66.38	42.54	97.87	2.60	0.85	6.98
North Korea	2.34%	1.19%	4.29%	49.47	31.94	71.78	1.16	0.38	3.08
Democratic Republic of the Congo	1.30%	0.65%	2.43%	42.64	25.78	63.34	0.55	0.17	1.54

Denmark	2.90%	1.47%	5.28%	25.80	16.59	37.65	0.75	0.24	1.99
Djibouti	0.83%	0.42%	1.55%	82.56	51.67	122.32	0.68	0.22	1.89
Dominica	1.25%	0.63%	2.35%	42.58	27.14	61.93	0.53	0.17	1.46
Dominican Republic	1.23%	0.62%	2.28%	51.00	32.20	75.56	0.63	0.20	1.73
Ecuador	0.57%	0.29%	1.05%	41.10	26.16	59.55	0.24	0.08	0.62
Egypt	1.75%	0.89%	3.22%	69.04	42.45	104.32	1.21	0.38	3.35
El Salvador	1.23%	0.62%	2.31%	38.99	24.20	56.60	0.48	0.15	1.31
Equatorial Guinea	1.45%	0.74%	2.71%	45.29	27.60	67.48	0.66	0.20	1.83
Eritrea	0.73%	0.36%	1.36%	58.14	36.36	84.79	0.42	0.13	1.16
Estonia	3.82%	1.96%	6.94%	49.85	31.53	71.96	1.90	0.62	4.99
Eswatini	1.43%	0.72%	2.67%	44.36	27.40	65.59	0.64	0.20	1.75
Ethiopia	0.82%	0.41%	1.53%	74.69	48.24	107.48	0.61	0.20	1.64
Fiji	2.75%	1.40%	5.03%	81.73	51.59	120.54	2.25	0.72	6.06
Finland	3.15%	1.60%	5.73%	52.80	34.23	75.45	1.66	0.55	4.32
France	3.09%	1.58%	5.64%	43.06	27.51	62.90	1.33	0.44	3.54
Gabon	1.42%	0.72%	2.64%	47.41	29.19	70.33	0.68	0.21	1.86
Gambia	1.50%	0.75%	2.76%	64.60	40.23	95.85	0.97	0.30	2.64
Georgia	3.35%	1.70%	6.13%	60.14	38.03	88.06	2.02	0.65	5.40
Germany	3.43%	1.75%	6.26%	58.49	37.25	85.23	2.01	0.65	5.34
Ghana	1.55%	0.78%	2.87%	44.99	27.73	67.33	0.70	0.22	1.93
Greece	2.75%	1.39%	5.06%	44.75	28.21	65.96	1.23	0.39	3.34
Greenland	4.43%	2.31%	7.92%	68.35	44.00	98.13	3.03	1.02	7.77
Grenada	1.21%	0.61%	2.25%	43.69	27.63	63.31	0.53	0.17	1.43
Guam	2.76%	1.40%	5.07%	97.64	61.69	142.63	2.69	0.86	7.23
Guatemala	1.21%	0.62%	2.30%	34.68	21.63	50.24	0.42	0.13	1.15
Guinea	1.39%	0.71%	2.61%	60.12	37.35	87.41	0.84	0.26	2.28
Guinea-Bissau	1.44%	0.73%	2.68%	53.48	32.83	78.77	0.77	0.24	2.11
Guyana	1.21%	0.61%	2.28%	42.76	26.73	63.05	0.52	0.16	1.44
Haiti	1.14%	0.58%	2.13%	37.67	23.47	55.43	0.43	0.14	1.18

Honduras	1.25%	0.63%	2.31%	38.09	23.89	55.30	0.48	0.15	1.27
Hungary	3.74%	1.91%	6.83%	54.67	34.74	79.64	2.05	0.66	5.44
Iceland	3.24%	1.66%	5.86%	45.95	29.40	67.39	1.49	0.49	3.95
India	2.67%	1.35%	4.86%	35.82	23.00	51.87	0.96	0.31	2.52
Indonesia	2.89%	1.46%	5.28%	79.91	50.58	116.45	2.31	0.74	6.15
Iran	1.83%	0.93%	3.36%	80.27	51.22	115.95	1.46	0.48	3.90
Iraq	1.89%	0.96%	3.47%	72.84	46.60	108.22	1.37	0.45	3.75
Ireland	3.30%	1.69%	5.99%	39.48	25.25	58.46	1.30	0.43	3.50
Israel	2.87%	1.46%	5.22%	30.27	19.43	43.87	0.87	0.28	2.29
Italy	2.72%	1.38%	4.96%	81.44	53.72	118.05	2.22	0.74	5.86
Jamaica	1.25%	0.63%	2.32%	49.09	30.87	71.67	0.61	0.19	1.66
Japan	1.11%	0.57%	2.04%	43.77	29.76	61.50	0.49	0.17	1.25
Jordan	1.78%	0.91%	3.28%	86.10	54.70	126.23	1.53	0.50	4.13
Kazakhstan	3.29%	1.68%	5.97%	44.19	28.20	63.42	1.45	0.47	3.79
Kenya	0.80%	0.40%	1.49%	83.44	53.29	119.71	0.67	0.21	1.78
Kiribati	2.55%	1.29%	4.74%	65.52	42.79	94.84	1.67	0.55	4.50
Kuwait	1.85%	0.94%	3.40%	90.05	56.76	131.65	1.66	0.53	4.48
Kyrgyzstan	3.05%	1.55%	5.53%	43.49	27.78	62.86	1.33	0.43	3.47
Laos	2.60%	1.32%	4.81%	50.25	31.68	73.44	1.31	0.42	3.53
Latvia	3.67%	1.89%	6.70%	50.75	32.19	73.93	1.86	0.61	4.96
Lebanon	1.86%	0.95%	3.44%	80.47	51.11	118.47	1.50	0.48	4.07
Lesotho	1.42%	0.72%	2.65%	39.51	24.45	57.72	0.56	0.17	1.53
Liberia	1.51%	0.76%	2.79%	62.73	38.54	93.13	0.95	0.29	2.60
Libya	1.81%	0.92%	3.33%	80.40	49.60	117.72	1.46	0.46	3.92
Lithuania	3.88%	1.98%	7.08%	45.23	28.88	65.18	1.76	0.57	4.61
Luxembourg	3.67%	1.86%	6.64%	69.19	44.33	99.25	2.54	0.82	6.59
Madagascar	0.78%	0.39%	1.45%	73.23	44.87	109.38	0.57	0.17	1.59
Malawi	0.82%	0.41%	1.54%	75.34	47.40	112.75	0.62	0.19	1.74
Malaysia	2.80%	1.43%	5.12%	72.61	45.58	105.75	2.03	0.65	5.42

Maldives	2.62%	1.33%	4.81%	63.06	39.17	93.31	1.65	0.52	4.49
Mali	1.42%	0.72%	2.65%	51.99	32.07	76.62	0.74	0.23	2.03
Malta	3.13%	1.60%	5.71%	66.56	41.80	98.71	2.09	0.67	5.64
Marshall Islands	2.53%	1.29%	4.66%	71.77	46.08	105.67	1.82	0.59	4.93
Mauritania	1.51%	0.77%	2.79%	63.48	39.24	94.43	0.96	0.30	2.63
Mauritius	2.67%	1.36%	4.88%	61.62	38.85	91.00	1.65	0.53	4.44
Mexico	1.37%	0.69%	2.54%	42.53	27.19	62.34	0.58	0.19	1.58
Federated States of Micronesia	2.46%	1.24%	4.54%	70.10	44.67	103.38	1.72	0.55	4.69
Monaco	3.13%	1.58%	5.72%	41.87	26.44	61.78	1.31	0.42	3.53
Mongolia	3.19%	1.62%	5.87%	48.25	30.48	70.83	1.54	0.50	4.16
Montenegro	3.48%	1.76%	6.34%	53.06	33.70	76.49	1.85	0.59	4.85
Morocco	1.82%	0.93%	3.33%	69.96	43.16	102.04	1.27	0.40	3.40
Mozambique	0.80%	0.40%	1.49%	63.38	39.38	93.26	0.51	0.16	1.39
Myanmar	2.56%	1.31%	4.67%	53.62	33.99	78.44	1.37	0.44	3.66
Namibia	1.44%	0.72%	2.67%	45.27	27.77	67.47	0.65	0.20	1.80
Nauru	2.66%	1.36%	4.91%	79.06	50.12	116.60	2.10	0.68	5.73
Nepal	2.37%	1.20%	4.30%	18.86	11.85	27.31	0.45	0.14	1.17
Netherlands	2.91%	1.48%	5.32%	35.69	22.54	52.47	1.04	0.33	2.79
New Zealand	4.46%	2.38%	7.82%	59.55	39.97	82.89	2.66	0.95	6.48
Nicaragua	1.27%	0.64%	2.38%	38.57	24.58	56.36	0.49	0.16	1.34
Niger	1.41%	0.71%	2.61%	51.95	32.61	78.10	0.73	0.23	2.04
Nigeria	1.58%	0.80%	2.90%	59.14	38.23	86.21	0.93	0.30	2.50
Niue	2.70%	1.38%	4.96%	87.72	56.21	126.82	2.37	0.77	6.29
Republic of North Macedonia	3.22%	1.62%	5.89%	50.13	31.33	73.27	1.61	0.51	4.32
Northern Mariana Islands	2.70%	1.37%	4.95%	86.19	56.26	123.68	2.32	0.77	6.12
Norway	3.04%	1.55%	5.53%	63.50	41.89	90.41	1.93	0.65	5.00
Oman	1.83%	0.94%	3.40%	69.04	43.11	99.42	1.27	0.40	3.38
Pakistan	2.66%	1.35%	4.85%	35.80	22.83	52.21	0.95	0.31	2.53

Palau	2.72%	1.39%	4.98%	81.68	52.20	119.61	2.22	0.73	5.96
Palestinian National Authority	1.73%	0.88%	3.20%	72.31	45.64	105.88	1.25	0.40	3.39
Panama	1.30%	0.65%	2.43%	39.08	24.70	57.37	0.51	0.16	1.39
Papua New Guinea	2.45%	1.24%	4.51%	69.80	44.82	101.70	1.71	0.55	4.58
Paraguay	1.70%	0.86%	3.15%	33.75	21.68	49.86	0.57	0.19	1.57
Peru	0.58%	0.29%	1.08%	39.00	24.34	56.47	0.22	0.07	0.61
Philippines	2.65%	1.34%	4.86%	70.56	45.00	102.68	1.87	0.60	4.99
Poland	3.80%	1.93%	6.85%	82.72	55.07	118.47	3.14	1.07	8.11
Portugal	3.12%	1.58%	5.74%	32.29	20.56	46.77	1.01	0.33	2.69
Puerto Rico	1.29%	0.65%	2.44%	54.17	34.90	78.85	0.70	0.23	1.93
Qatar	1.83%	0.94%	3.37%	75.48	48.43	111.75	1.38	0.45	3.76
South Korea	1.31%	0.67%	2.41%	30.84	19.77	44.74	0.40	0.13	1.08
Moldova	3.68%	1.88%	6.71%	48.88	31.68	69.65	1.80	0.60	4.68
Romania	3.38%	1.71%	6.17%	62.26	40.40	91.34	2.10	0.69	5.63
Russia	3.70%	1.91%	6.72%	51.61	33.77	73.69	1.91	0.64	4.95
Rwanda	0.78%	0.39%	1.45%	60.33	37.31	89.62	0.47	0.15	1.30
Saint Kitts and Nevis	1.26%	0.63%	2.35%	44.07	27.78	63.86	0.55	0.18	1.50
Saint Lucia	1.25%	0.63%	2.31%	46.25	29.47	66.89	0.58	0.19	1.55
Saint Vincent and the Grenadines	1.25%	0.63%	2.33%	47.40	30.22	70.03	0.59	0.19	1.63
Samoa	2.72%	1.38%	4.98%	84.03	53.61	122.54	2.29	0.74	6.11
San Marino	3.12%	1.58%	5.65%	44.07	28.23	64.61	1.38	0.45	3.65
São Tomé and Príncipe	1.62%	0.82%	3.00%	63.75	40.03	94.24	1.03	0.33	2.83
Saudi Arabia	1.73%	0.88%	3.18%	70.23	43.89	102.43	1.21	0.39	3.26
Senegal	1.48%	0.74%	2.75%	72.40	45.58	106.44	1.07	0.34	2.93
Serbia	3.19%	1.60%	5.86%	38.49	26.07	54.83	1.23	0.42	3.21
Seychelles	2.73%	1.40%	4.96%	64.65	40.97	96.52	1.77	0.57	4.78
Sierra Leone	1.58%	0.80%	2.94%	62.55	38.71	92.74	0.99	0.31	2.73
Singapore	1.39%	0.72%	2.57%	29.90	19.80	43.16	0.42	0.14	1.11

Slovakia	3.84%	1.96%	6.99%	67.23	43.25	95.05	2.58	0.85	6.65
Slovenia	3.39%	1.71%	6.23%	61.07	41.06	86.76	2.07	0.70	5.40
Solomon Islands	2.48%	1.26%	4.55%	71.23	45.64	103.58	1.77	0.57	4.71
Somalia	0.74%	0.37%	1.40%	63.01	38.76	94.01	0.47	0.14	1.31
South Africa	1.62%	0.82%	2.99%	62.72	40.18	91.73	1.02	0.33	2.74
South Sudan	0.79%	0.40%	1.48%	71.79	44.11	106.37	0.57	0.17	1.58
Spain	3.09%	1.58%	5.59%	41.40	26.18	60.32	1.28	0.41	3.37
Sri Lanka	2.64%	1.35%	4.85%	66.15	41.22	96.50	1.74	0.56	4.68
Sudan	1.78%	0.90%	3.27%	70.69	44.11	103.37	1.26	0.40	3.38
Suriname	1.23%	0.62%	2.30%	49.61	31.66	73.22	0.61	0.20	1.68
Sweden	4.58%	2.36%	8.24%	90.70	57.40	132.98	4.15	1.36	10.95
Switzerland	2.64%	1.35%	4.84%	37.81	25.00	53.32	1.00	0.34	2.58
Syria	1.77%	0.91%	3.29%	69.38	43.25	103.69	1.23	0.39	3.41
Taiwan	2.36%	1.24%	4.18%	56.35	37.04	80.45	1.33	0.46	3.37
Tajikistan	2.99%	1.53%	5.44%	49.77	31.46	72.08	1.49	0.48	3.92
Thailand	2.59%	1.32%	4.71%	58.05	37.15	84.50	1.50	0.49	3.98
Timor-Leste	2.52%	1.27%	4.60%	56.11	35.65	80.84	1.41	0.45	3.72
Togo	1.44%	0.72%	2.68%	58.01	35.59	85.74	0.84	0.26	2.30
Tokelau	2.68%	1.36%	4.93%	85.28	54.43	125.32	2.29	0.74	6.18
Tonga	2.76%	1.39%	5.05%	86.17	55.26	122.51	2.38	0.77	6.19
Trinidad and Tobago	1.29%	0.65%	2.39%	50.11	31.83	73.18	0.65	0.21	1.75
Tunisia	1.76%	0.89%	3.24%	74.67	47.63	109.18	1.32	0.42	3.54
Turkey	1.85%	0.93%	3.39%	61.06	40.74	87.02	1.13	0.38	2.95
Turkmenistan	3.19%	1.61%	5.82%	49.43	31.56	71.89	1.58	0.51	4.19
Tuvalu	2.63%	1.33%	4.85%	78.60	49.91	115.36	2.07	0.66	5.59
Uganda	0.77%	0.39%	1.44%	57.95	35.87	85.38	0.45	0.14	1.23
Ukraine	3.86%	1.99%	7.05%	49.11	31.63	70.34	1.90	0.63	4.96
United Arab Emirates	1.82%	0.94%	3.35%	78.54	49.60	116.60	1.43	0.46	3.91
United Kingdom	3.90%	2.08%	6.84%	37.51	25.01	53.39	1.46	0.52	3.65

Tanzania	0.92%	0.47%	1.73%	76.69	47.87	112.64	0.71	0.22	1.95
United States	4.73%	2.62%	8.05%	108.41	72.39	151.98	5.13	1.89	12.24
United States Virgin Islands	1.24%	0.63%	2.31%	45.25	29.14	65.65	0.56	0.18	1.52
Uruguay	1.97%	1.00%	3.67%	58.40	37.26	85.42	1.15	0.37	3.14
Uzbekistan	3.01%	1.53%	5.47%	47.73	30.37	69.61	1.44	0.47	3.81
Vanuatu	2.76%	1.39%	5.07%	77.55	49.83	112.32	2.14	0.69	5.69
Venezuela	1.32%	0.67%	2.46%	40.81	25.33	60.37	0.54	0.17	1.49
Vietnam	2.78%	1.41%	5.07%	59.84	38.04	86.67	1.67	0.54	4.39
Yemen	1.65%	0.84%	3.04%	67.34	42.05	99.77	1.11	0.35	3.03
Zambia	0.77%	0.39%	1.43%	68.36	42.02	101.47	0.53	0.16	1.45
Zimbabwe	1.41%	0.71%	2.62%	44.62	27.41	65.70	0.63	0.20	1.72

PAF – population attributable fraction
LL – lower limit of 95% uncertainty interval
UL – upper limit of 95% uncertainty interval
UI – uncertainty interval
YLD – years lived with disability
